# Supplementary material for: Durable lymph-node expansion is associated with the efficacy of therapeutic vaccination
Source: Nat Biomed Eng. 2024 May 6;8(10):1226–42. doi: 10.1038/s41551-024-01209-3 (PMC11485260; doi:10.1038/s41551-024-01209-3)
Supplement: Supplementary file 1 — Supplementary figures and tables. [file 41551_2024_1209_MOESM1_ESM.pdf]

nature biomedical engineering

Article

https://doi.org/10.1038/s41551-024-01209-3

Durable lymph-node expansion is associated with the efficacy of therapeutic vaccination

In the format provided by the authors and unedited

Contents

Supplementary Fig. 1 l MPS vaccination induces robust, prolonged LN expansion.
Supplementary Fig. 2 l F-actin distribution in LNs.
Supplementary Fig. 3 l Mapping LN viscoelasticity.
Supplementary Fig. 4 l LN mechanical properties vary with location.
Supplementary Fig. 5 l The LN mechanical response to immunization depends on tissue location.
Supplementary Fig. 6 l LN density after immunization.
Supplementary Fig. 7 l Representative gating strategy to identify immune cell subsets.
Supplementary Fig. 8 l Immune and stromal cell populations in LNs after vaccination.
Supplementary Fig. 9 l scRNAseq gating, cluster identification, and population frequencies.
Supplementary Fig. 10. cDC2s are transcriptionally altered after MPS vaccination.
Supplementary Fig. 11 l scRNAseq gating analysis of *Cd274* (PD-L1) expression.
Supplementary Fig. 12 l Plasma cells are enriched and express mature Ig after MPS vaccination.
Supplementary Fig. 13 l MPS vaccination expands inflammatory monocytes.
Supplementary Fig. 14 l Inflammatory monocyte depletion.
Supplementary Fig. 15 l Therapeutic study to assess correlations of LN expansion with vaccine efficacy.
Supplementary Fig. 16 l Vaccine therapeutic efficacy.
Supplementary Fig. 17 l The adaptive, antitumour vaccine response correlates with LN expansion.
Supplementary Fig. 18 l Antigen-free MPS “jump-start” strategy boosts bolus vaccine response.
Supplementary Fig. 19 l Determining optimal timing of “jump-start” strategy to improve vaccine response.
Supplementary Fig. 20. Therapeutic “jump-start” experiment layout.
Supplementary Fig. 21 l Booster vaccination expands lymph nodes and improves adaptive immune responses.

Supplementary Table 1 l Antibodies utilized in flow cytometry
Supplementary Table 2 l Antibodies utilized in lymph node immunohistochemistry

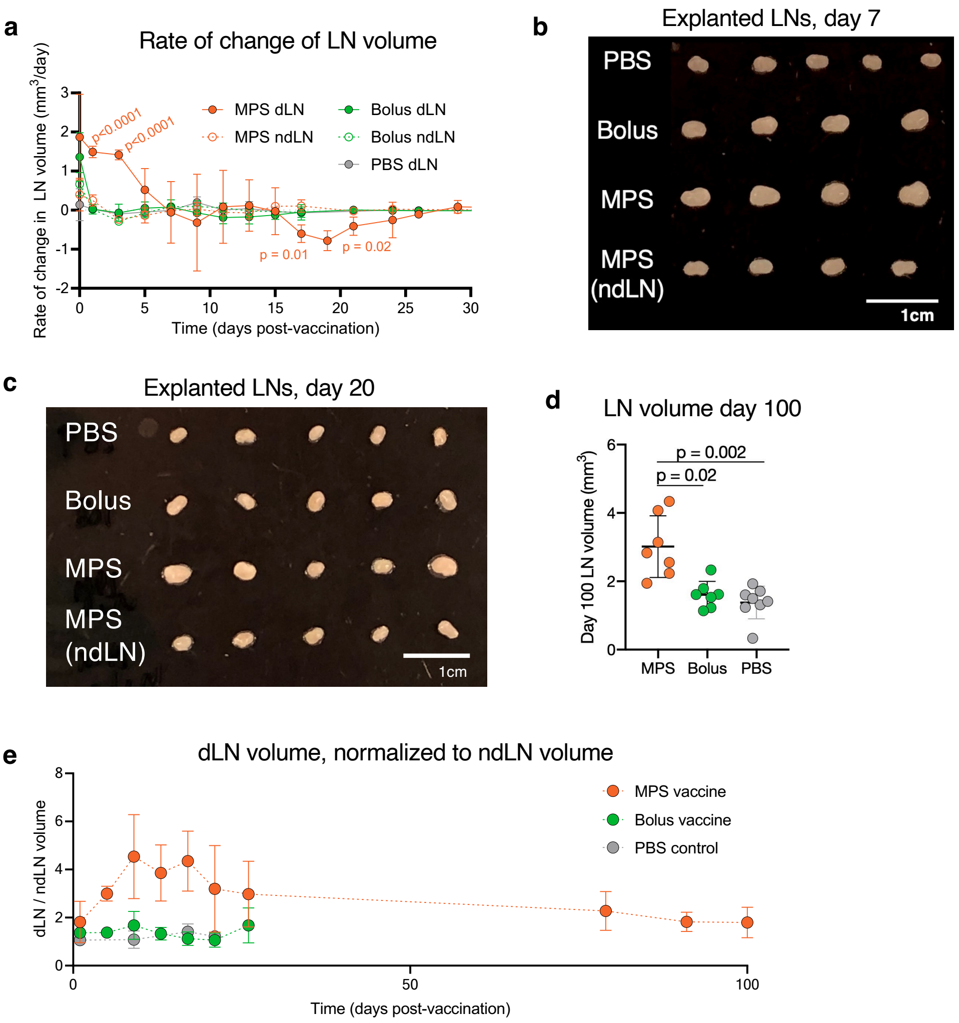

Supplementary Fig. 1 I MPS vaccination induces robust, prolonged LN expansion. Mice were immunized with MPS or bolus vaccines delivering GM-CSF, CpG, and OVA protein, and compared to PBS-injected controls. Vaccine-draining and non-draining LNs were longitudinally imaged using high frequency ultrasound. n = 7 (MPS and bolus) or 8 (PBS) biologically independent animals per group, imaged longitudinally in two cohorts. (a) Rate of change of LN volume over time. Statistical analysis was performed using analysis of variance (ANOVA) with Tukey's post hoc test. Only differences between one group and all other groups are shown. (b) Photograph of LNs explanted 7 days after vaccination. Each LN was derived from a unique mouse. (c) Photograph of LNs explanted 20 days after vaccination. Each LN was derived from a unique mouse. (d) LN volumes 100 days after immunization. n = 7 (MPS and bolus) or 8 (PBS) biologically independent animals per group. Statistical analysis was performed using a Kruskal-Wallis test with Dunn's post hoc test. (e) Draining LN volume over time, normalized to the volume of the contralateral non-draining LN in the same mouse. n = 4 biologically independent animals per group. For a, c, and e means depicted; error bars, s.d.

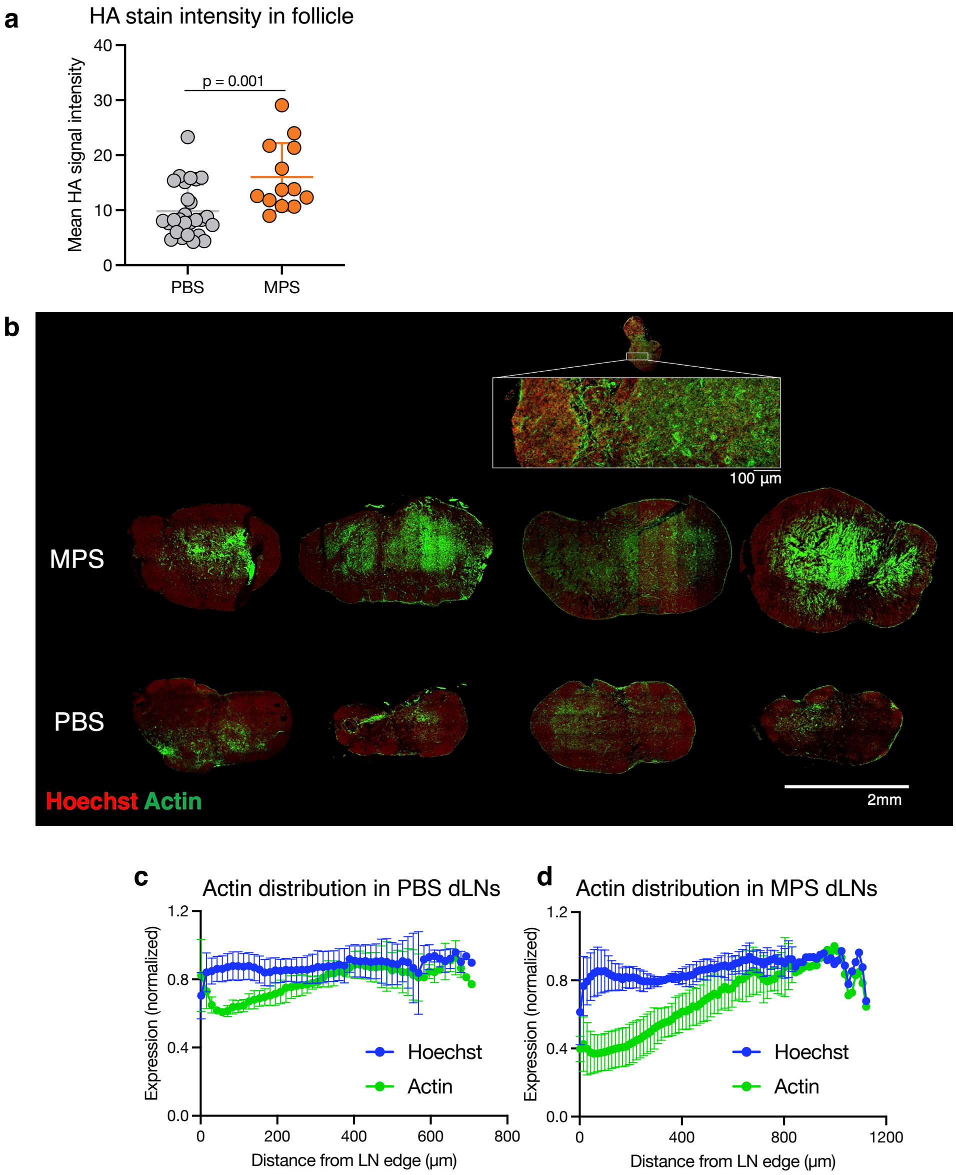

Supplementary Fig. 2 I F-actin distribution in LNs. Mice were treated with MPS vaccines (GM-CSF, CpG, OVA) or PBS and LNs were harvested after 7 days. (a) ImageJ quantification of hyaluronic acid stain intensity in follicles from PBS or MPS draining LNs on day 20. Statistical analysis was performed using a Mann-Whitney test. n = 27 follicles across 5 biologically independent animals (PBS) or 13 follicles across 3 biologically independent animals (MPS). (b) IHC of LNs on day 7 stained for F-actin; one MPS and PBS LN from this image were selected for Fig. 2c. n = 4 biologically independent animals per group. Distribution of nuclear (Hoechst) and F-actin stains across PBS (c) and MPS (d) draining LNs (from periphery to center), quantified through a custom MATLAB code, on day 7. Values are normalized to the highest signal in each LN. For a and c-d, means are depicted; error bars, s.d. For c-d, n = 4 (PBS) or 3 (MPS) biologically independent animals per group.

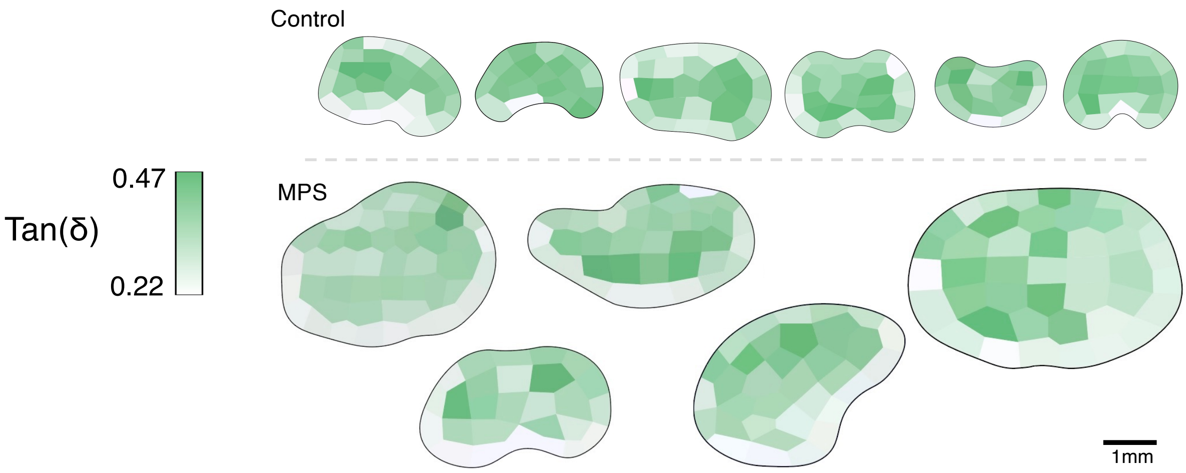

Supplementary Fig. 3 | Mapping LN viscoelasticity. Mice were treated with MPS vaccines (GM-CSF, CpG, OVA) or PBS and LNs were harvested after 7 days. Heatmaps depicting tan(δ) across individual LNs, scaled low-high within each LN (mean low value = 0.22; mean high value = 0.47). Scale bar = 1mm.

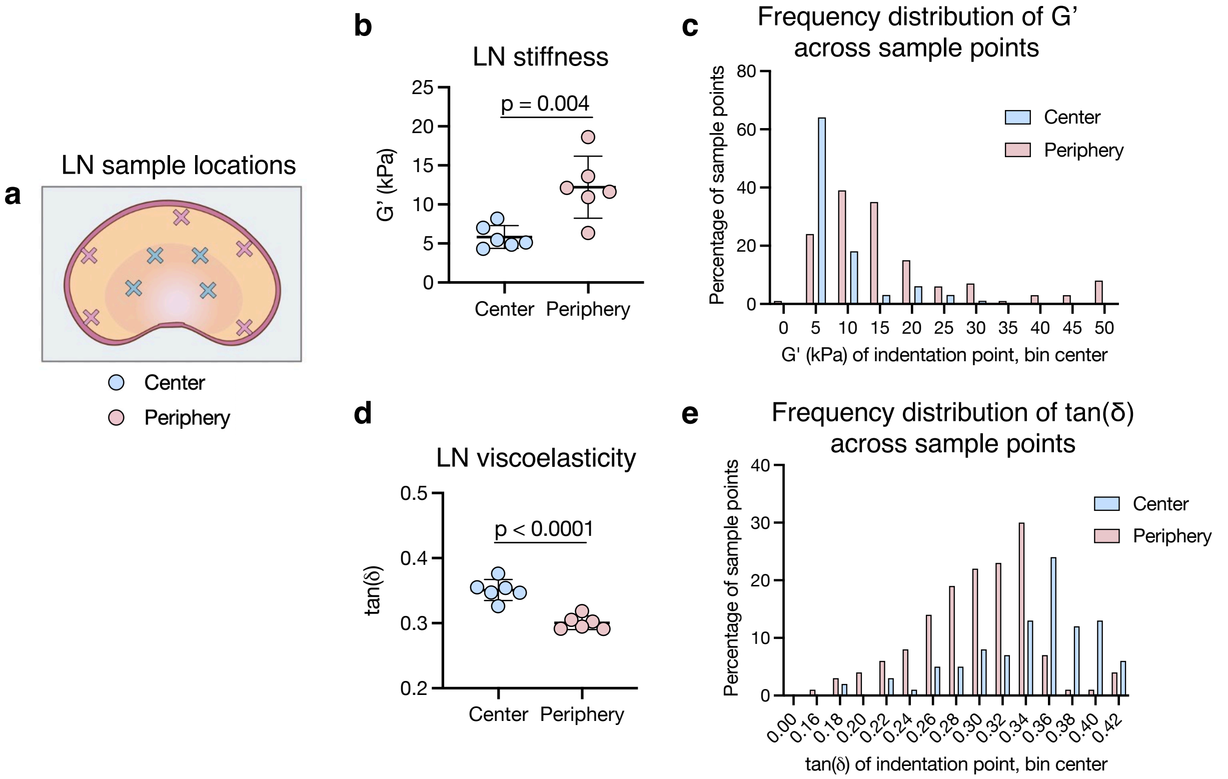

Supplementary Fig. 4 I LN mechanical properties vary with location. Inguinal LNs were collected from naïve mice for nanoindentation, and kept hydrated throughout the procedure. (a) Schematic depicting example sample locations chosen on the center or periphery. (b) Mean G' of sample points collected in the center or periphery across LNs. Each data point represents a unique LN. (c) Frequency distribution of G' of individual samples points taken across control (naïve or PBS) LNs from multiple experiments. (d) Mean tan(δ) of sample points collected in the center or periphery across LNs. Each data point represents a unique LN. (e) Frequency distribution of tan(δ) of individual samples points taken across control (naïve or PBS) LNs from multiple experiments. n = 6 independent LNs; means are depicted; error bars, s.d. For b and d, statistical analysis was performed using a two-tailed t test.

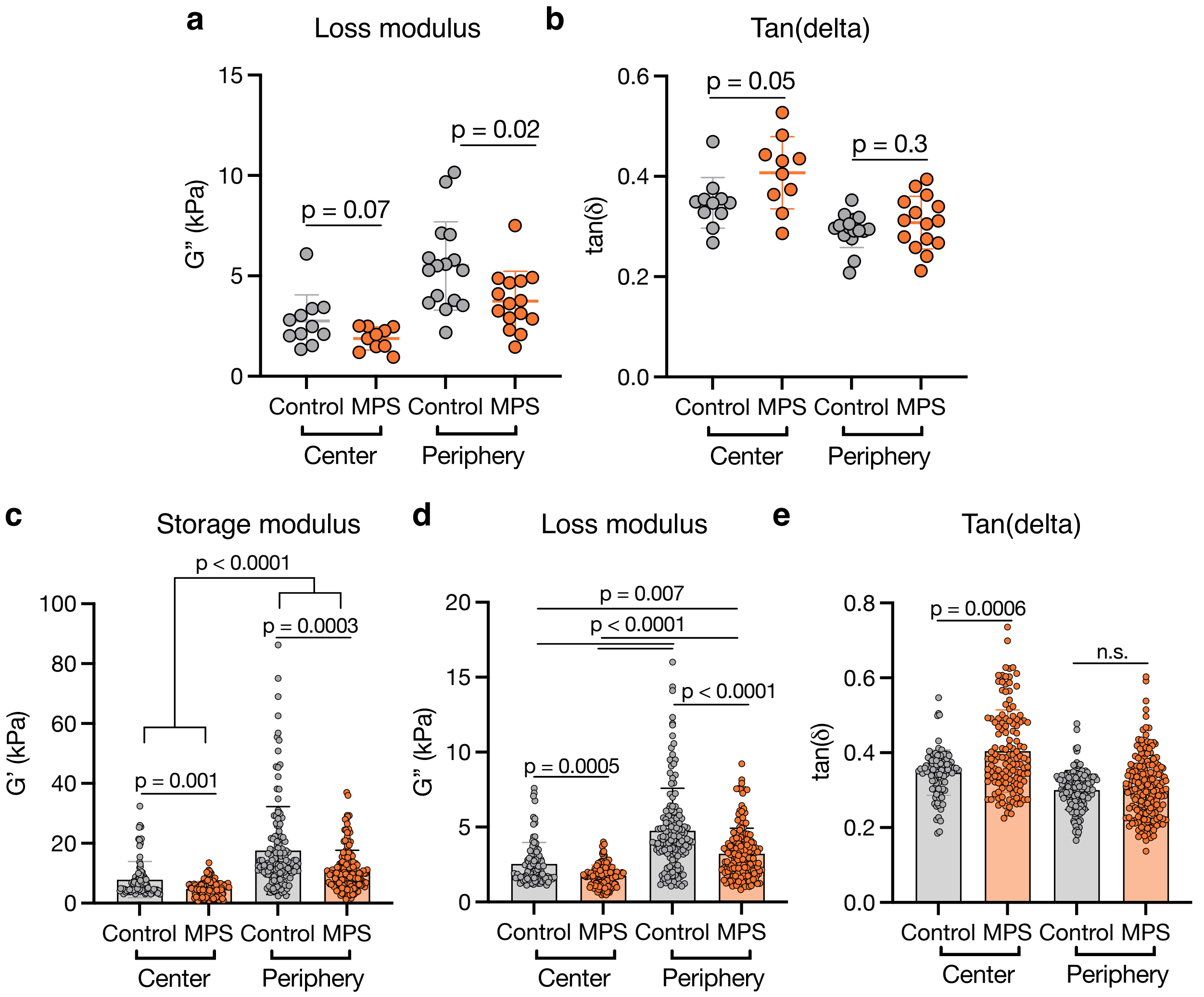

Supplementary Fig. 5 l The LN mechanical response to immunization depends on tissue location. Mice were immunized with MPS vaccines (containing GM-CSF, CpG, OVA) and dLNs were collected on day 7 for nanoindentation, and compared to PBS-injected mice or naïve controls. Mean (a) G'', and (b) tan(δ) of sample points across each LN. For a-b, each data point represents a unique LN/mouse; n = 11 (control, center), 10 (MPS, center), 16 (control, periphery), and 15 (MPS, periphery) biologically independent animals per group. Individual sample points plotted for (c) G', (d) G'', and (e) tan(δ) collected from LNs in parts (a-c). For c-e, each data point represents a single nanoindentation location, with multiple across individual LNs. For a-e, results are combined from three independent experiments; means depicted; error bars, s.d. Statistical analysis was performed using a Mann-Whitney test (a-b (center) and e), two-tailed t test (a-b (periphery)), and Kruskal-Wallis test with Dunn's post hoc test (c-d).

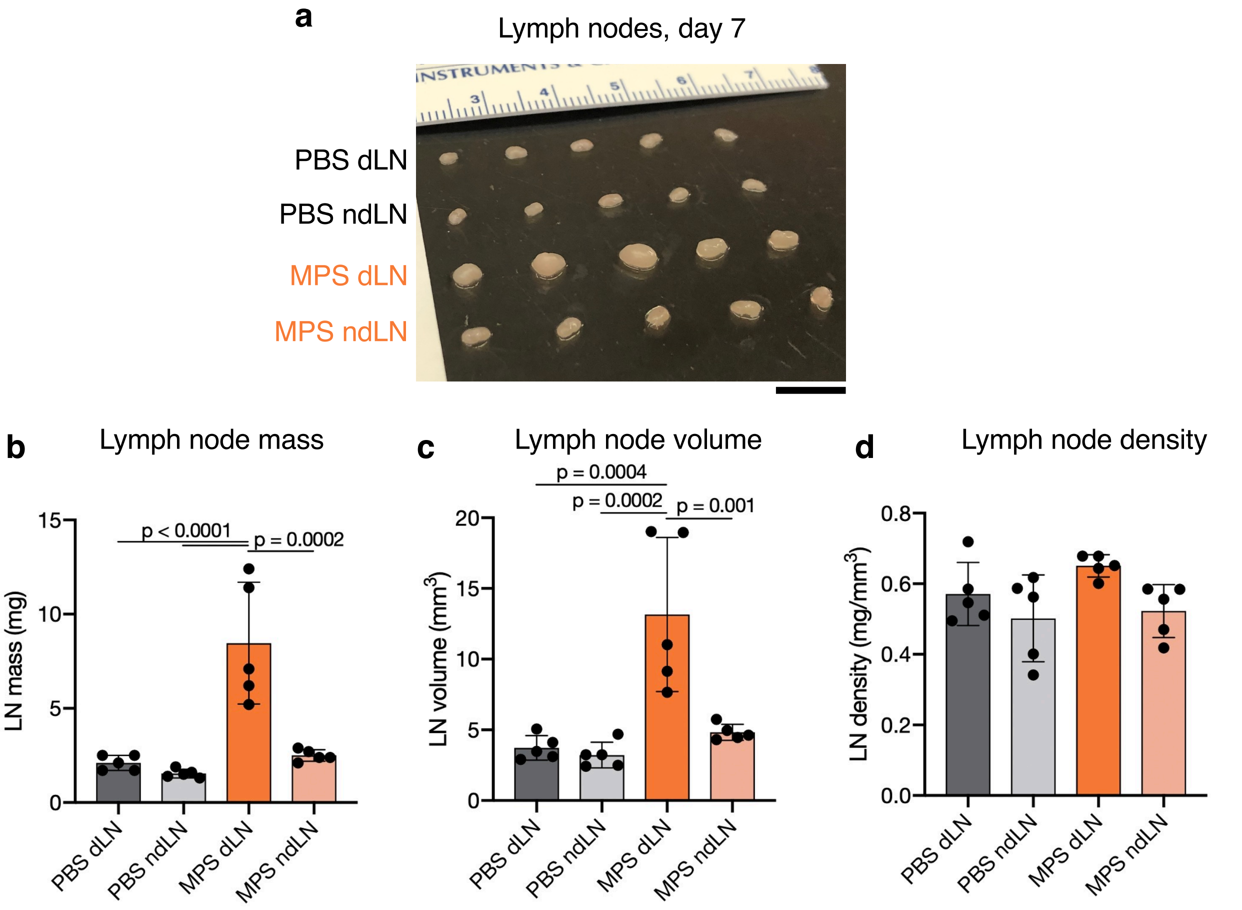

Supplementary Fig. 6 I LN density after immunization. Mice were immunized with MPS vaccines containing GM-CSF, CpG, and OVA protein and compared to control mice injected with PBS. Inguinal dLNs and ndLNs (contralateral to vaccine injection site) were harvested after 7 days. (a) Photograph of explanted LNs at day 7. LN mass (b), volume (c), and density (calculated as mass divided by volume) (d). For d, no statistically significant difference was found between groups. n = 5 biologically independent animals per group (MPS or PBS); means depicted; error bars, s.d. Statistical analysis was performed using analysis of variance (ANOVA) with Tukey's post hoc test. Scale bar = 1cm.

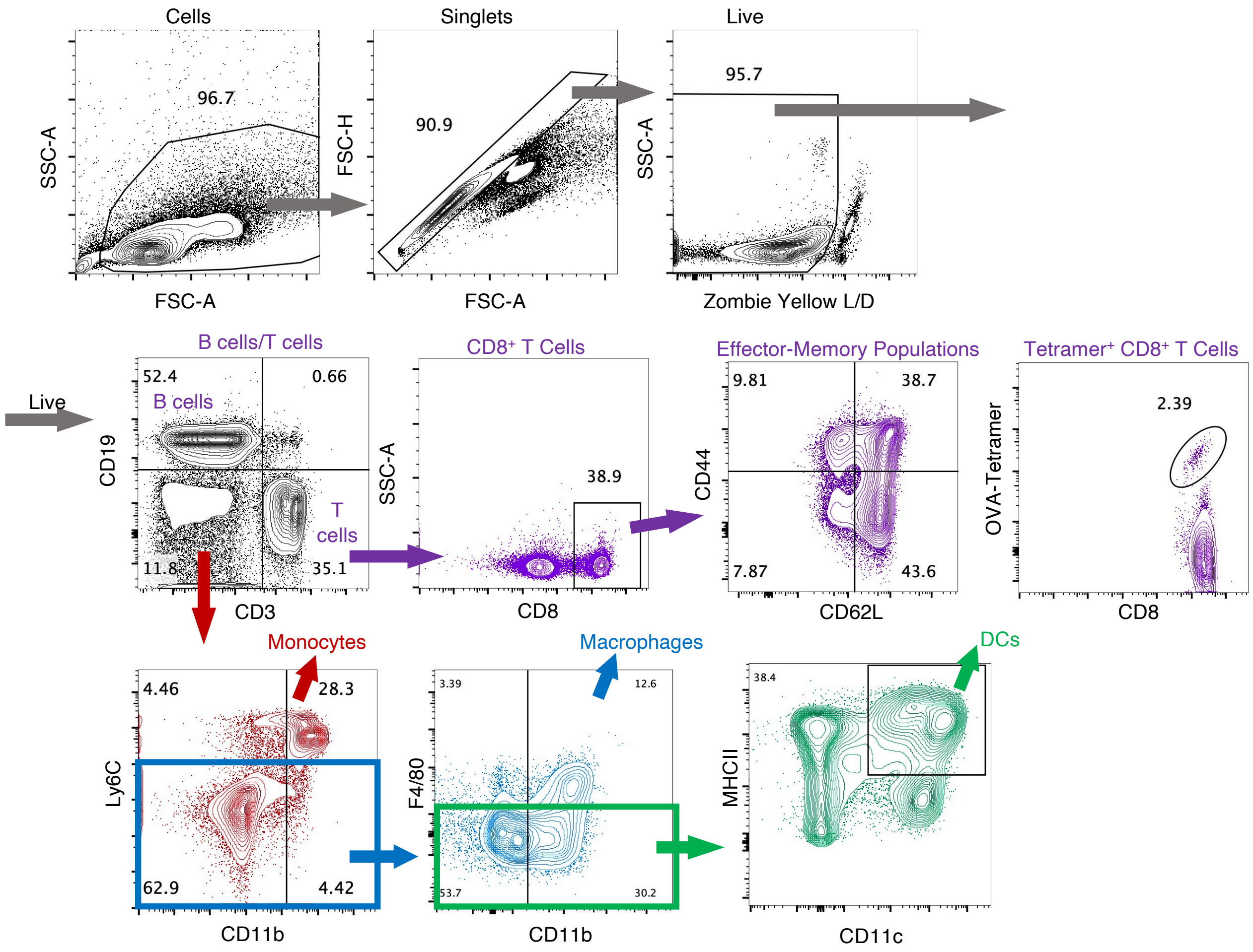

Supplementary Fig. 7 I Representative gating strategy to identify immune cell subsets.

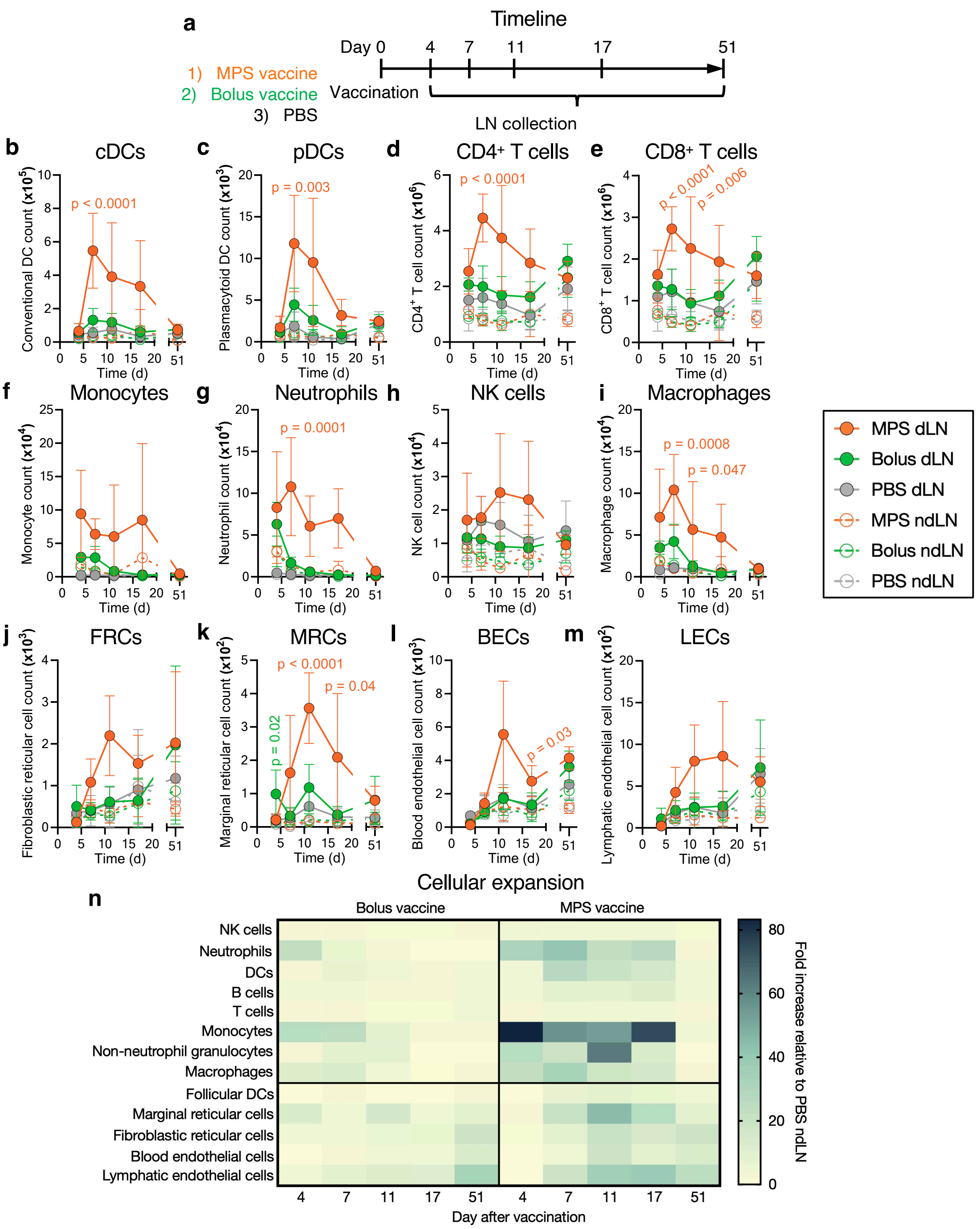

Supplementary Fig. 8 | Immune and stromal cell populations in LNs after vaccination. Mice were immunized with MPS or bolus vaccines containing GM-CSF, CpG, and OVA protein, euthanized on days 4, 7, 11, 17, and 51 for LN harvest and analysis through flow cytometry, and compared to PBS-injected controls. Immune and stromal populations were analyzed through flow cytometry over time. (a) Experimental timeline. (b) Conventional DCs (CD3$^-$ B220$^-$ NK1.1$^-$ Ly6G$^-$ Ly6C$^-$ CD64$^-$ F4/80$^-$ CD11c$^+$ MHCII$^+$), (c) plasmacytoid DCs (CD3$^-$ B220$^+$ CD11c$^+$ CD11b$^-$ Ly6C$^+$ Siglec-H$^+$), (d) CD4$^+$ T cells (CD3$^+$ B220$^-$ CD4$^+$ CD8$^-$), (e) CD8$^+$ T cells (CD3$^+$ B220$^-$ CD8$^+$ CD4$^-$), (f) monocytes (CD3$^-$ B220$^-$ NK1.1$^-$ Ly6G$^-$ CD11b$^+$ Ly6C$^+$), (g) neutrophils (CD3$^-$ B220$^-$ NK1.1$^-$ CD49b$^-$ CD11b$^+$ CD11c$^-$ SSC-A^{int} Ly6G^{hi}), (h) natural killer cells (CD3$^-$ B220$^-$ NK1.1$^+$ CD49b$^+$), (i) macrophages (CD3$^-$ B220$^-$ NK1.1$^-$ Ly6G$^-$ Ly6C$^-$ CD64$^+$ F4/80$^+$), (j) fibroblastic reticular cells (CD45$^-$ CD31$^-$ PDPN$^+$ CD21/35$^-$ MAdCAM-1$^-$), (k) marginal reticular cells (CD45$^-$ CD31$^-$ PDPN$^+$ CD21/35$^-$ MAdCAM-1$^+$), (l) blood endothelial cells (CD45$^-$ CD31$^+$ PDPN$^-$), and (m) lymphatic endothelial cells (CD45$^-$ CD31$^+$ PDPN$^+$) in LNs. (n) Heatmap of fold cellular expansion, relative to the PBS control, of the indicated cell types over time (days labeled below) after immunization with the bolus vaccine (left) or MPS vaccine (right). Mean cellular expansion was calculated relative to the PBS ndLN condition at day 4. For b-n, n = 4

(MPS dLN days 4, 11 and MPS ndLN day 7) or 5 (all other timepoints and groups) biologically independent animals per group per timepoint; means depicted; error bars, s.d. Statistical analysis was performed using analysis of variance (ANOVA) with Tukey's post hoc test for normally distributed samples, and a Kruskal-Wallis test with Dunn's post hoc test otherwise; statistical significance is shown between the MPS dLN group and all other groups.

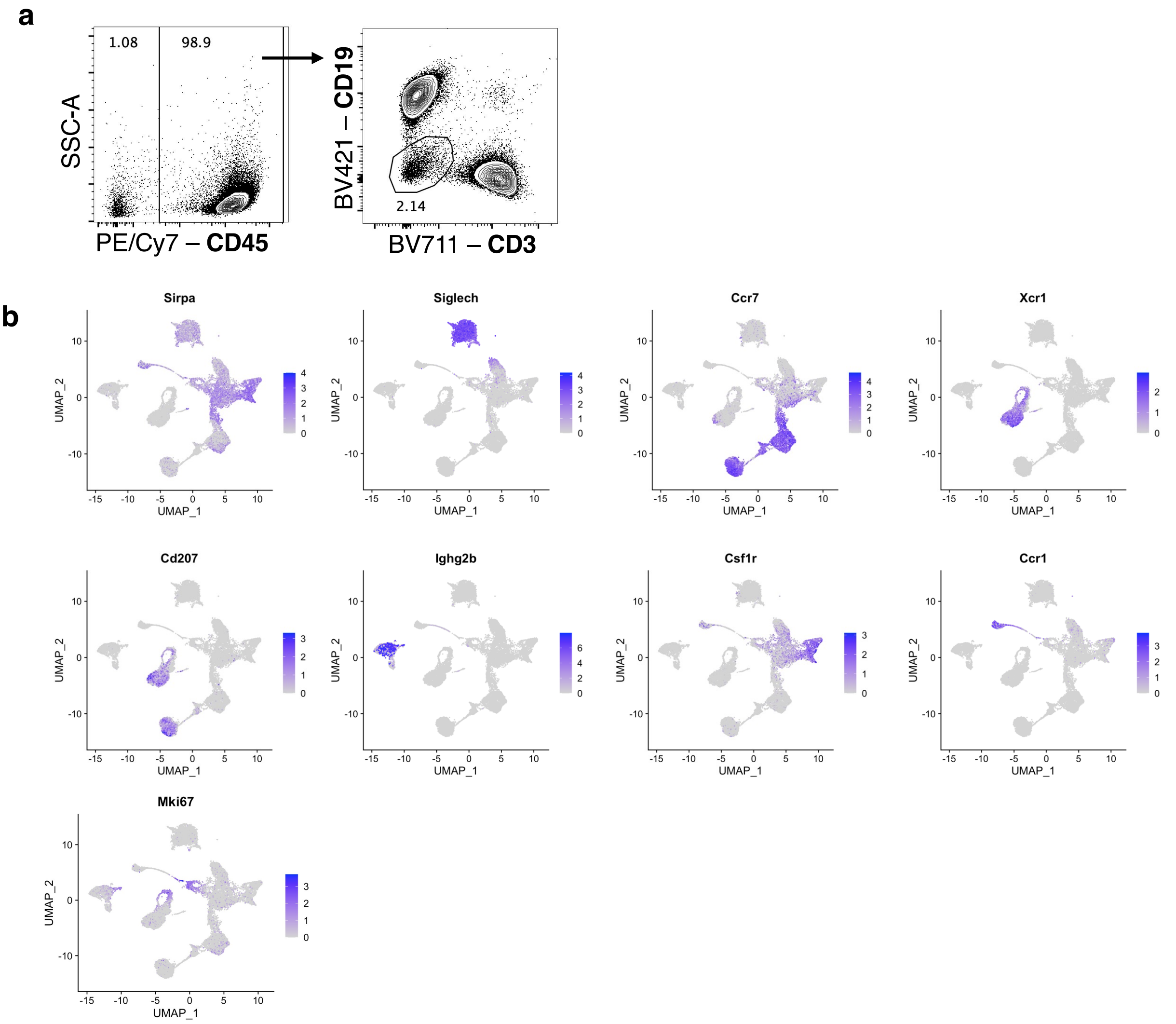

Supplementary Fig. 9 I scRNAseq gating, cluster identification, and population frequencies. (a) Representative flow cytometry plots depicting FACS-sorting strategy to identify myeloid cells ($CD45^{+}CD3^{-}$ $CD19^{-}$) for scRNAseq analysis. (b) UMAP of cells across conditions from Fig. 3h, with marker gene expression distinguishing the identified clusters (color scaled for each gene as indicated). n = 5 biologically independent animals per group (15 total among MPS, bolus, and naive).

a

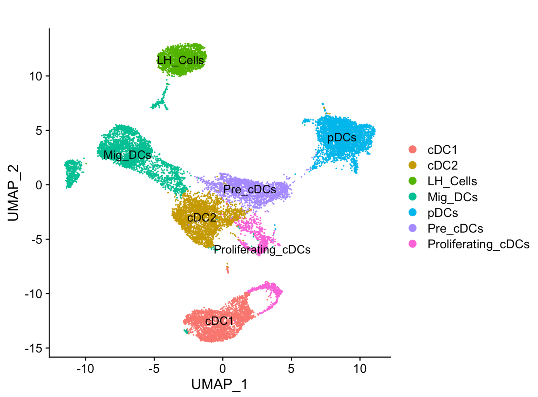

b

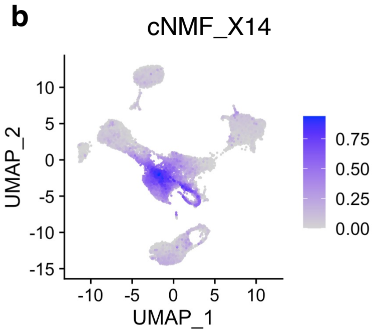

c

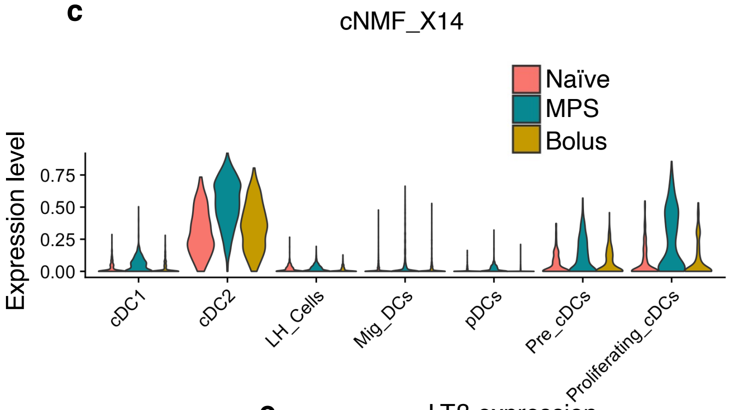

d

	cDC2 program
Rank	cNMF_X14
1	Ffar2
2	Cass4
3	Rasgef1b
4	Il1r2
5	Elmo1
6	Fam105a
7	Limd2
8	Stap1
9	Sirpb1a
10	Ltb
11	Ppp1r14a
12	Egr2

13	Gm1966
14	Gm6377
15	Btg2
16	Ifitm6
17	Chn2
18	Lst1
19	Cd86
20	Cyp4f16
21	Bbs9
22	Sirpa
23	Elmsan1
24	Clec4a2
25	Sirpb1b

e

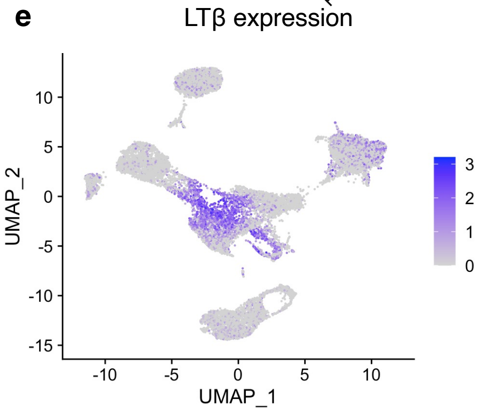

Supplementary Fig. 10. cDC2s are transcriptionally altered after MPS vaccination. (a) UMAP of dendritic cell populations from Fig. 3h. (b) Expression of cNMF X14 (cDC2-associated gene module) among DC populations, color scaled as indicated. (c) Expression level of cNMF X14 gene module among DC subsets. (d) Top 25 genes associated with cNMF X14 gene module. (e) Expression of *Ltb* among DC subsets. For a-e, n = 5 biologically independent animals per group (15 total among MPS, bolus, and naive).

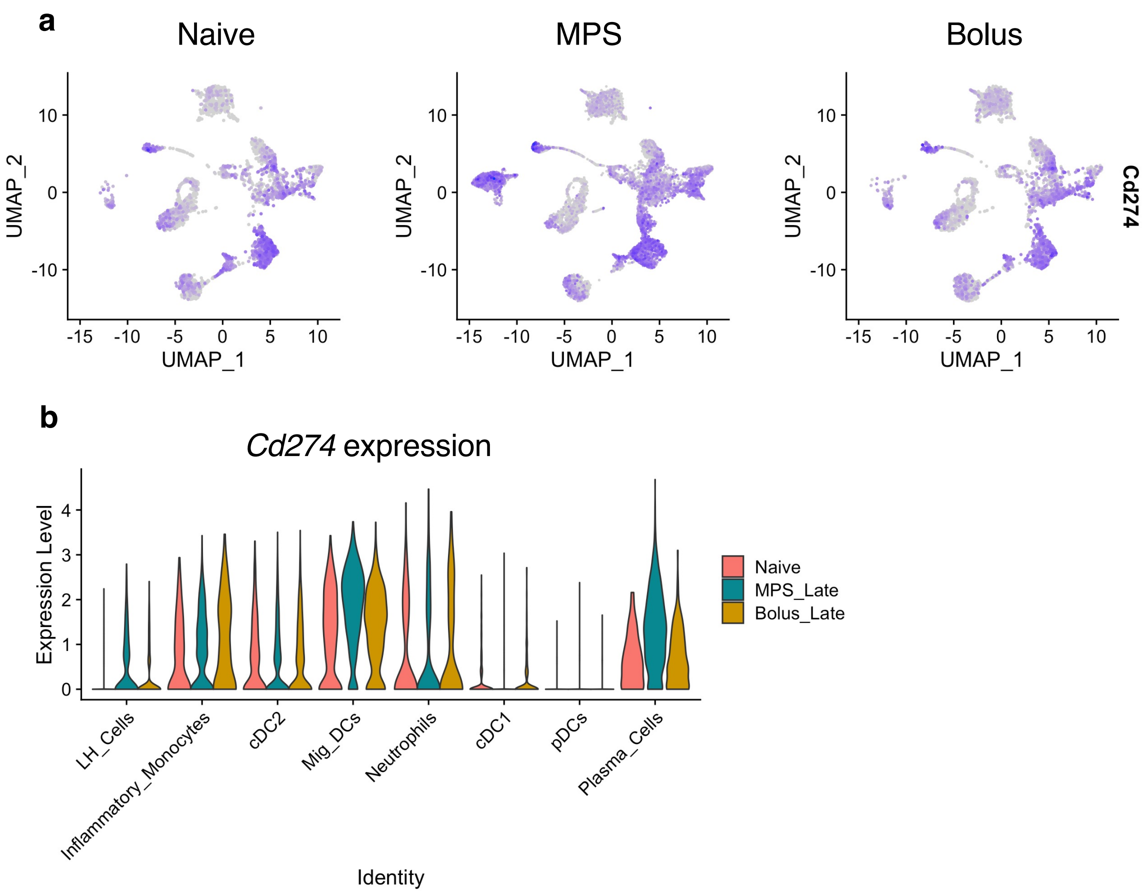

Supplementary Fig. 11 I scRNAseq gating analysis of *Cd274* (PD-L1) expression. (a) Expression of *Cd274* (PD-L1) among cells across conditions from Fig. 3h. (b) Expression level of *Cd274* among myeloid cell subsets. For a-b, n = 5 biologically independent animals per group (15 total among MPS, bolus, and naive).

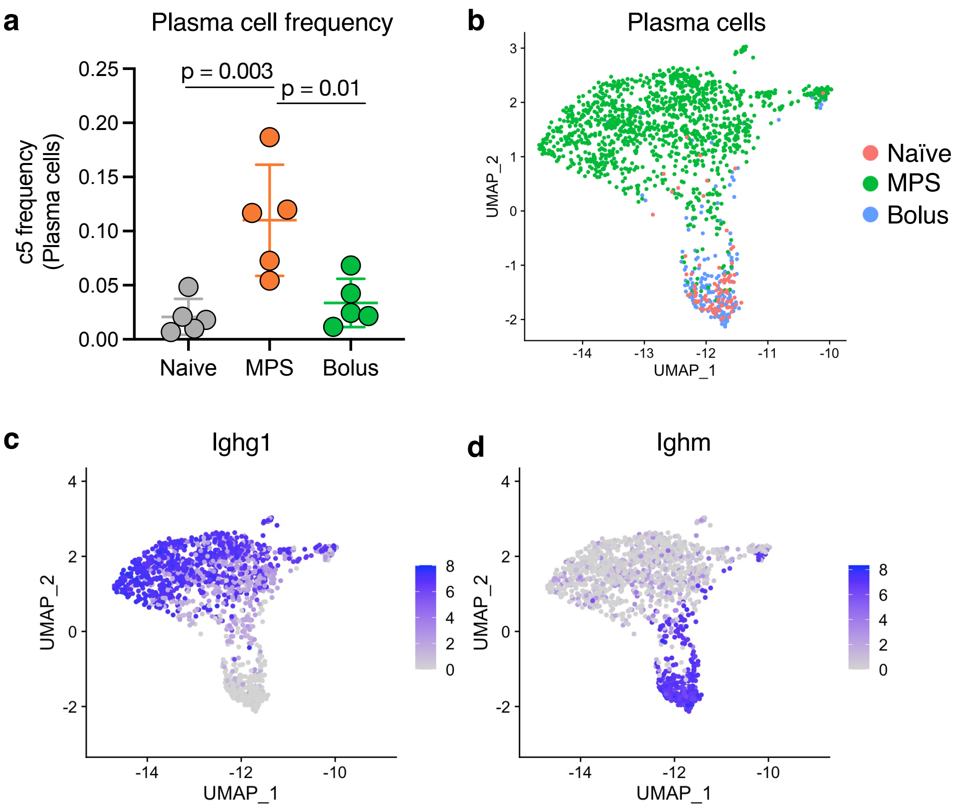

Supplementary Fig. 12 | Plasma cells are enriched and express mature Ig after MPS vaccination. (a) Frequency of plasma cells among different conditions. Means depicted; error bars, s.d. (b) UMAP of plasma cells from Fig. 3h. Expression of (c) *Ighg1* and (d) *Ighm* among plasma cells, color scaled as indicated. For a-d, n = 5 biologically independent animals per group (15 total among MPS, bolus, and naive).

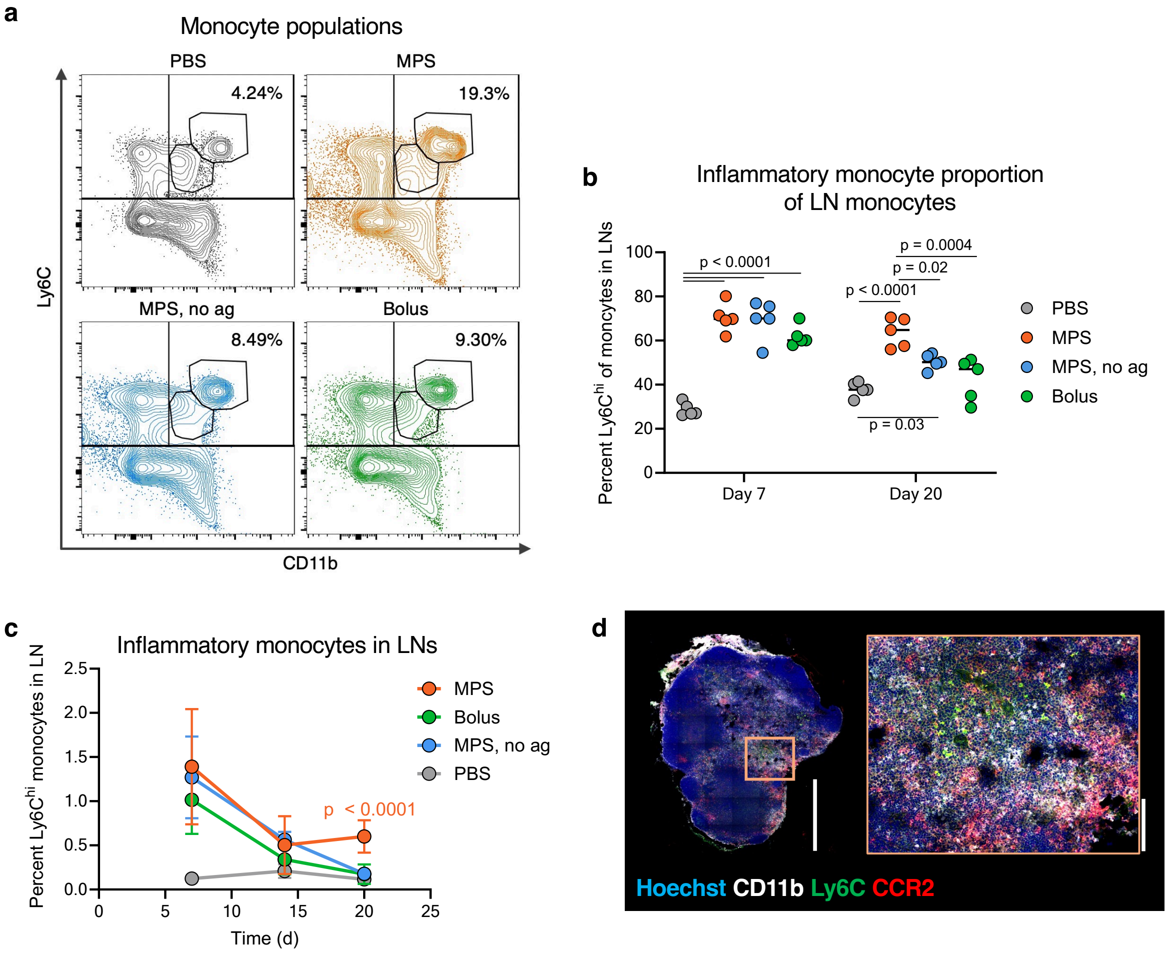

Supplementary Fig. 13 | MPS vaccination expands inflammatory monocytes. Mice were treated with MPS or bolus vaccines (containing GM-CSF, CpG, OVA), MPS vaccine without antigen (GM-CSF, CpG only), or PBS, and LNs were collected on days 7, 14, and 20 for cellular analysis. n = 5 biologically independent animals per group per timepoint. (a) Representative flow cytometry plots depicting CD3$^-$ CD19$^-$ cells from LNs on day 20. Top right quadrant signifies monocytes (CD11b$^+$ Ly6C$^+$); upper population in that quadrant signifies inflammatory Ly6C^{hi} monocytes. (b) Proportion Ly6C^{hi} of monocytes (CD3$^-$ CD19$^-$CD11b$^+$Ly6C$^+$) in LNs on days 7 and 20. (c) Ly6C^{hi} inflammatory monocyte proportions in the LN over time. (d) IHC image depicting an MPS-vaccinated mouse LN extracted on day 20 and stained for inflammatory monocyte markers. Scale bars = 0.5mm (left), 100µm (right). For b-c, statistical analyses were performed using analysis of variance (ANOVA) with Tukey's post hoc test; means depicted; error bars, s.d. For c, only differences present between one group and all other groups are shown.

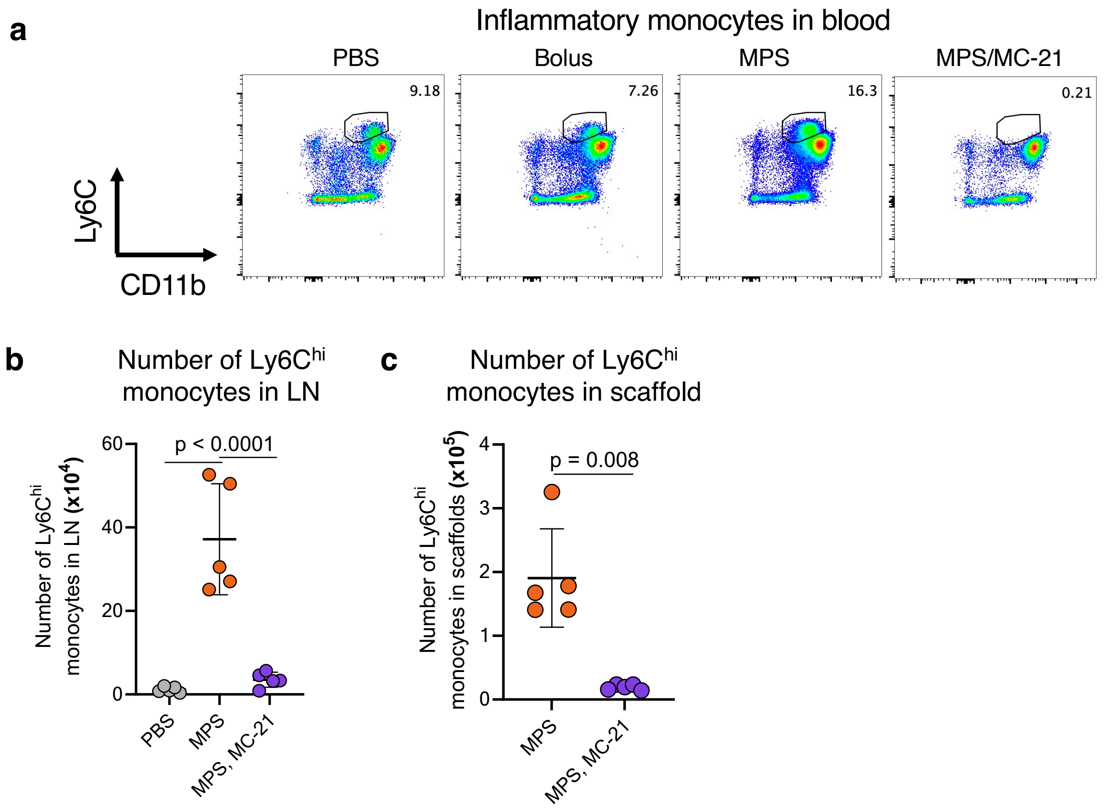

Supplementary Fig. 14 l Inflammatory monocyte depletion. Mice were administered MPS vaccines (containing GM-CSF, CpG, OVA) or PBS. One group of MPS-vaccinated mice was treated with MC-21 CCR2-depleting mAb daily from days 1-5 ("MC-21 expansion") and one group was treated daily from days 10-14 ("MC-21 maintenance"), according to the timeline in Fig. 5f. Peripheral blood was collected on days 6, 8, 14, and 20 for cellular analysis. (a) Representative flow cytometry plots depicting $CD3^-$ $B220^-$ cells in blood on day 14. At this time, the $Ly6C^{hi}$ monocyte population was depleted in the MPS/MC-21 maintenance group (depicted). Numbers of $Ly6C^{hi}$ monocytes in LNs (b) and MPS scaffolds (c) on day 5 of mice given MPS vaccines and treated with or without MC-21 daily from days 0-4. For b, statistical analysis was performed using analysis of variance (ANOVA) with Tukey's post hoc test. For c, statistical analysis was performed using a Mann-Whitney test. For b-c, means depicted; error bars, s.d.; n = 5 biologically independent animals per group.

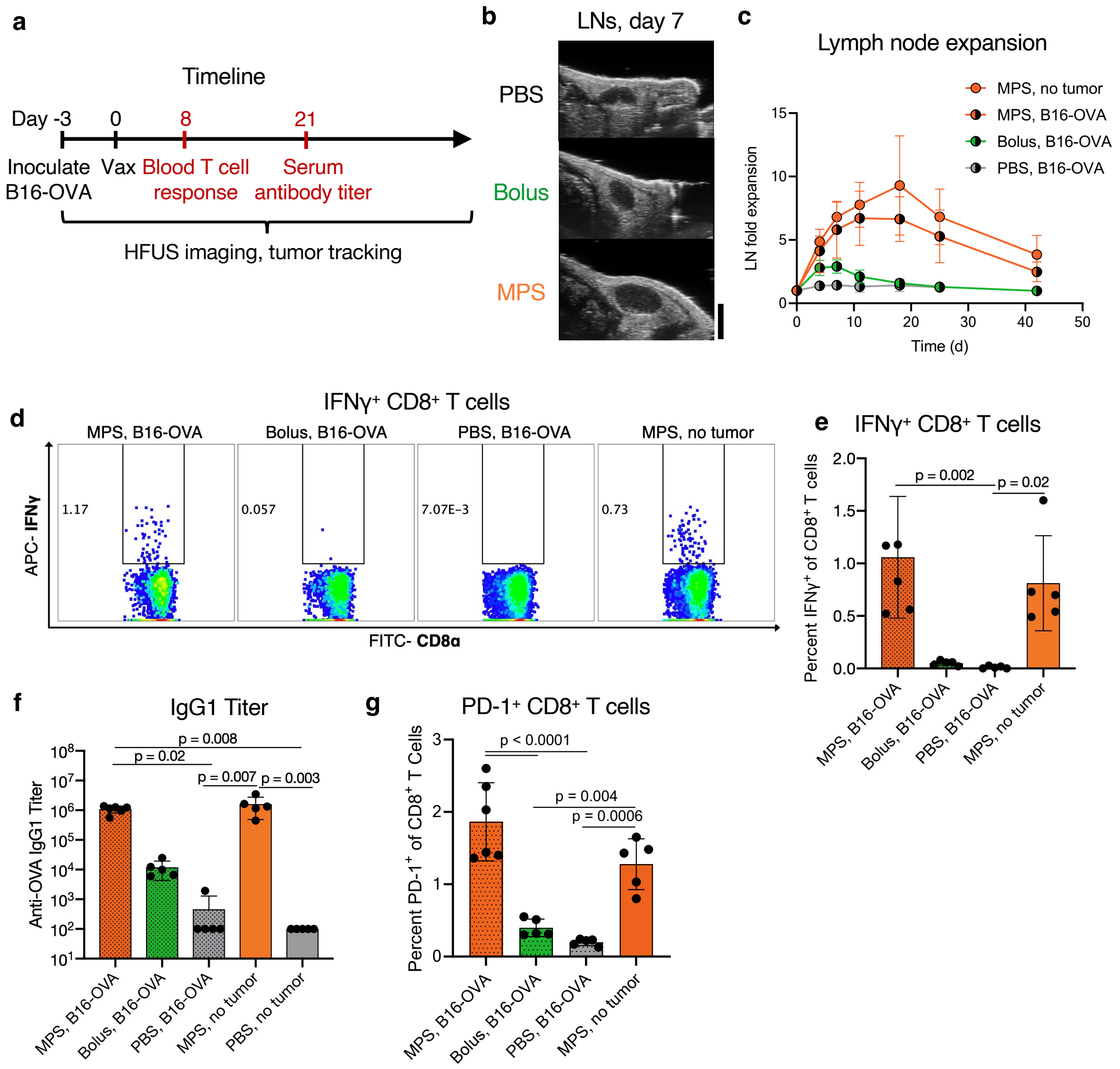

Supplementary Fig. 15 I Therapeutic study to assess correlations of LN expansion with vaccine efficacy. Mice were inoculated with B16-OVA tumours and three days later treated with MPS or bolus vaccines containing GM-CSF, CpG, and OVA protein, and compared to PBS-injected controls. A fourth group of tumour-free mice was treated with MPS vaccines (called “MPS, no tumour”). Inguinal dLNs were imaged using HFUS at multiple timepoints, and blood was collected 8 and 21 days after vaccination to assess T cell responses and serum antibody titers, respectively. n = 5-6 biologically independent animals per group. (a) Experimental timeline. (b) Representative images of dLNs 7 days after vaccination. Scale bar = 2mm. (c) LN fold expansion over time, relative to the day 0 timepoint. Values are normalized to the baseline volume for each individual LN. No PBS-injected mice survived at day 42. (d) Representative flow cytometry plots of IFNγ+ CD8+ T cells after SIINFEKL peptide restimulation. (e) Proportion IFNγ+ of CD8+ T cells in blood after SIINFEKL peptide restimulation. Statistical analysis was performed using a Kruskal-Wallis test with Dunn’s post hoc test. (f) Serum anti-OVA IgG1 antibody titer, 21 days after immunization. Statistical analysis was performed using a Kruskal-Wallis test with Dunn’s post hoc test. (g) PD-1 expression on CD8+ T cells. Statistical analysis was performed using analysis of variance (ANOVA) with Tukey’s post hoc test. For c and e-g, means are depicted; error bars, s.d.

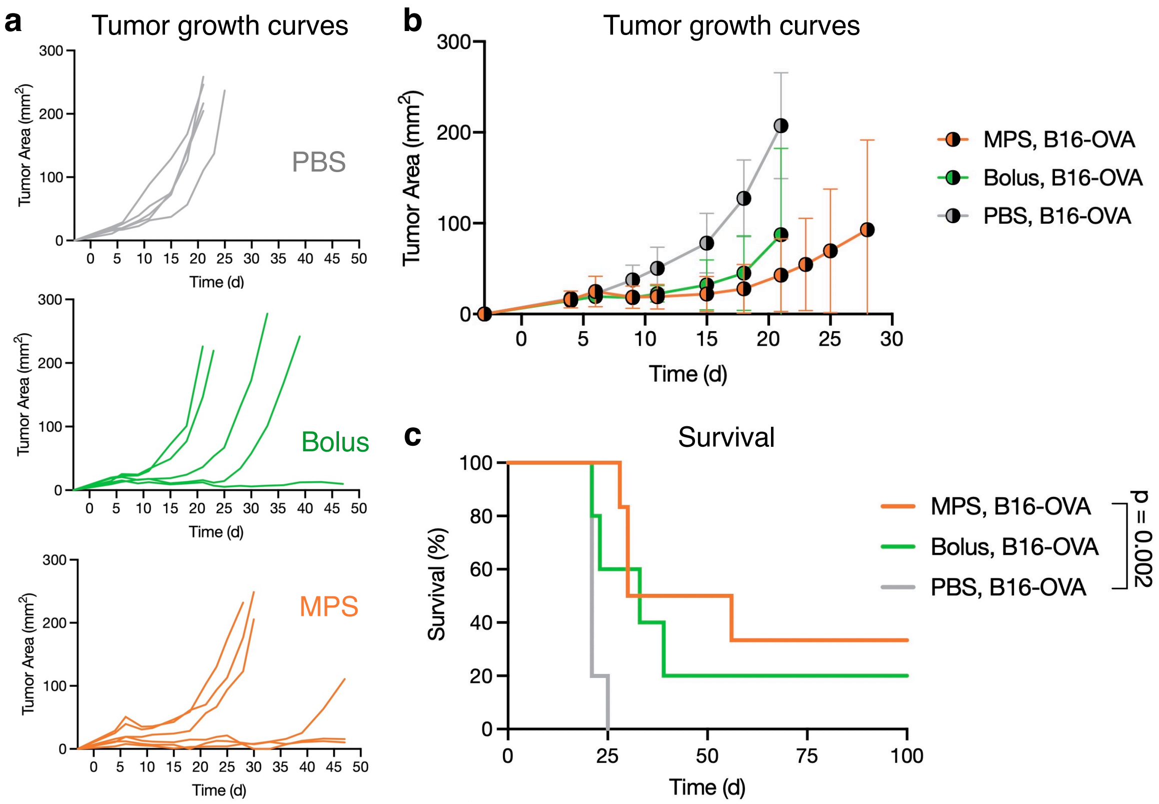

Supplementary Fig. 16 | Vaccine therapeutic efficacy. Mice were treated as described in Supplementary Fig. 4. Tumour growth was tracked externally using calipers and mice were euthanized at humane endpoints. (a) Individual growth curves of treated mice. (b) Combined growth curves. (c) Kaplan-Meier curves depicting survival of groups. n = 5 (bolus and PBS) or 6 (MPS) biologically independent animals per group; statistical analysis was performed using a log-rank (Mantel-Cox) test, correcting for multiple comparisons

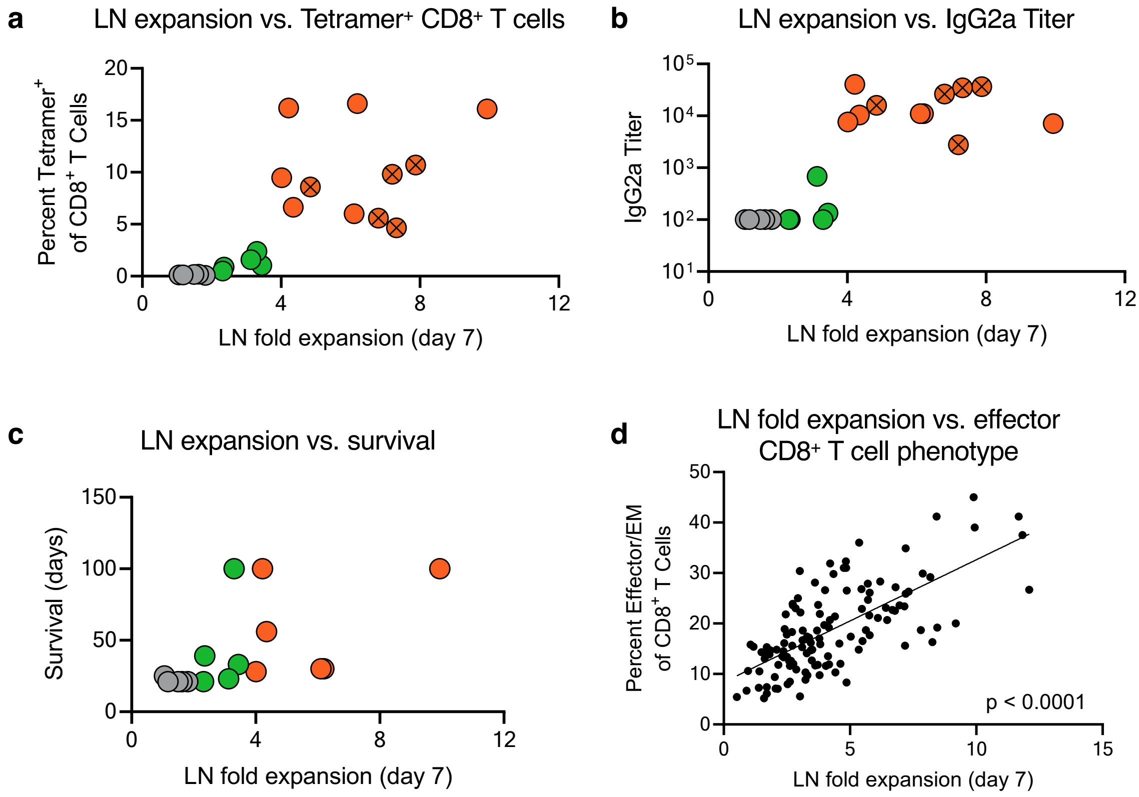

Supplementary Fig. 17 I The adaptive, antitumour vaccine response and LN expansion. LN expansion from Fig. 6b is plotted against vaccine response data from Fig. 6 and Supplementary Fig. 18. LN fold expansion 7 days after vaccination is plotted against (a) OVA-tetramer+ CD8+ T cells, (b) anti-OVA IgG2a titers, and (c) long-term survival. For a-b, n = 21 biologically independent animals. For c, n = 16 biologically independent animals. (d) LN fold expansion 7 days after vaccination is plotted against the proportion of effector (CD44+CD62L-) CD8+ T cells in the blood 8 days after vaccination. n = 125 biologically independent animals combined from 6 independent experiments; linear regression was performed and results are statistically significant ($p < 0.0001$).

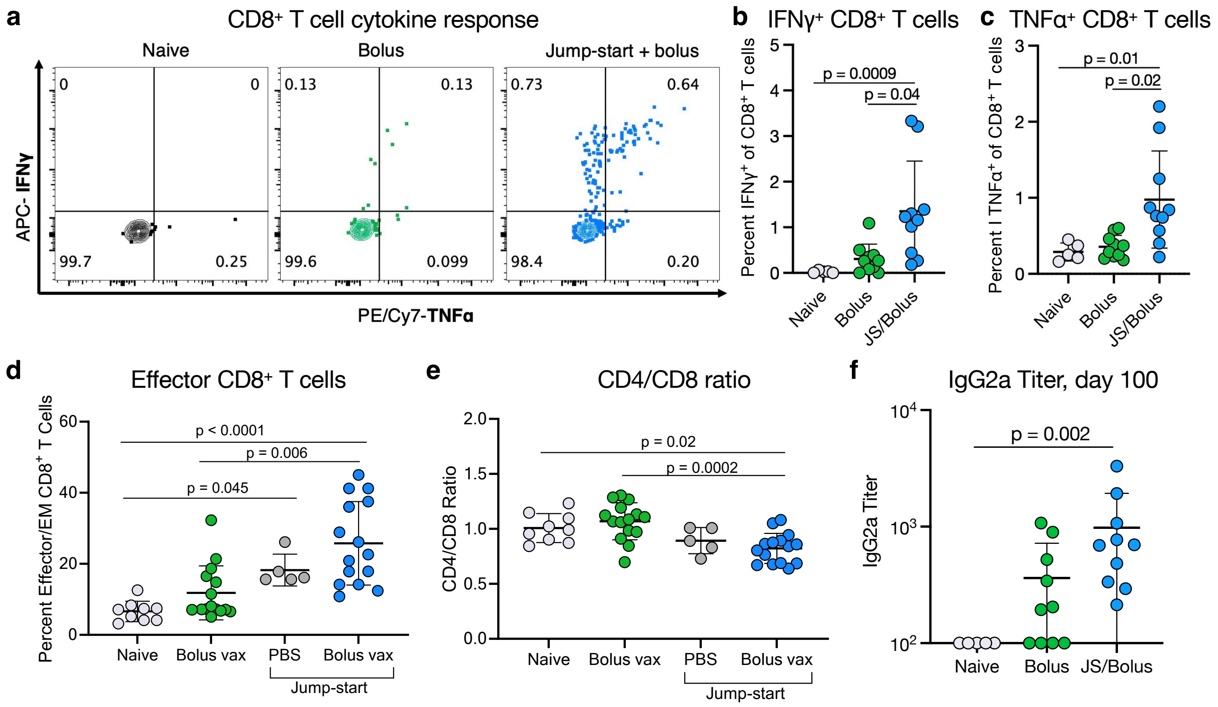

Supplementary Fig. 18 I Antigen-free MPS "jump-start" strategy boosts bolus vaccine response. Mice were injected with PBS or a bolus vaccine on day 0, or injected with an MPS no-antigen "jump-start" on day -7 followed by PBS or a bolus vaccine (GM-CSF, CpG, and OVA protein) on day 0. Mice were bled after 8 and 100 days for T cell analysis and serum antibody titers, respectively. (a) Representative flow cytometry plots depicting $CD8^+$ T cell cytokine production after restimulation with OVA SIINFEKL peptide. Quantification of IFNγ (b) and TNFα production (c). (d) Proportion of effector-phenotype ($CD44^+CD62L^-$) $CD8^+$ T cells in the blood. Statistical analysis was performed using a Kruskal-Wallis test with Dunn's post hoc test. (e) CD4/CD8 T cell ratio in the blood. Statistical analysis was performed using analysis of variance (ANOVA) with Tukey's post hoc test. (f) Anti-OVA IgG2a titer at 100 days. For b-c and f, n = 5 (naïve) or 10 (other groups) biologically independent animals per group; statistical analysis was performed using a Kruskal-Wallis test with Dunn's post hoc test. For d-e, n = 5 (jump-start + PBS), 10 (naïve), or 15 (other groups) biologically independent animals per group; results are combined from two independent experiments. For b-e, means depicted; error bars, s.d.

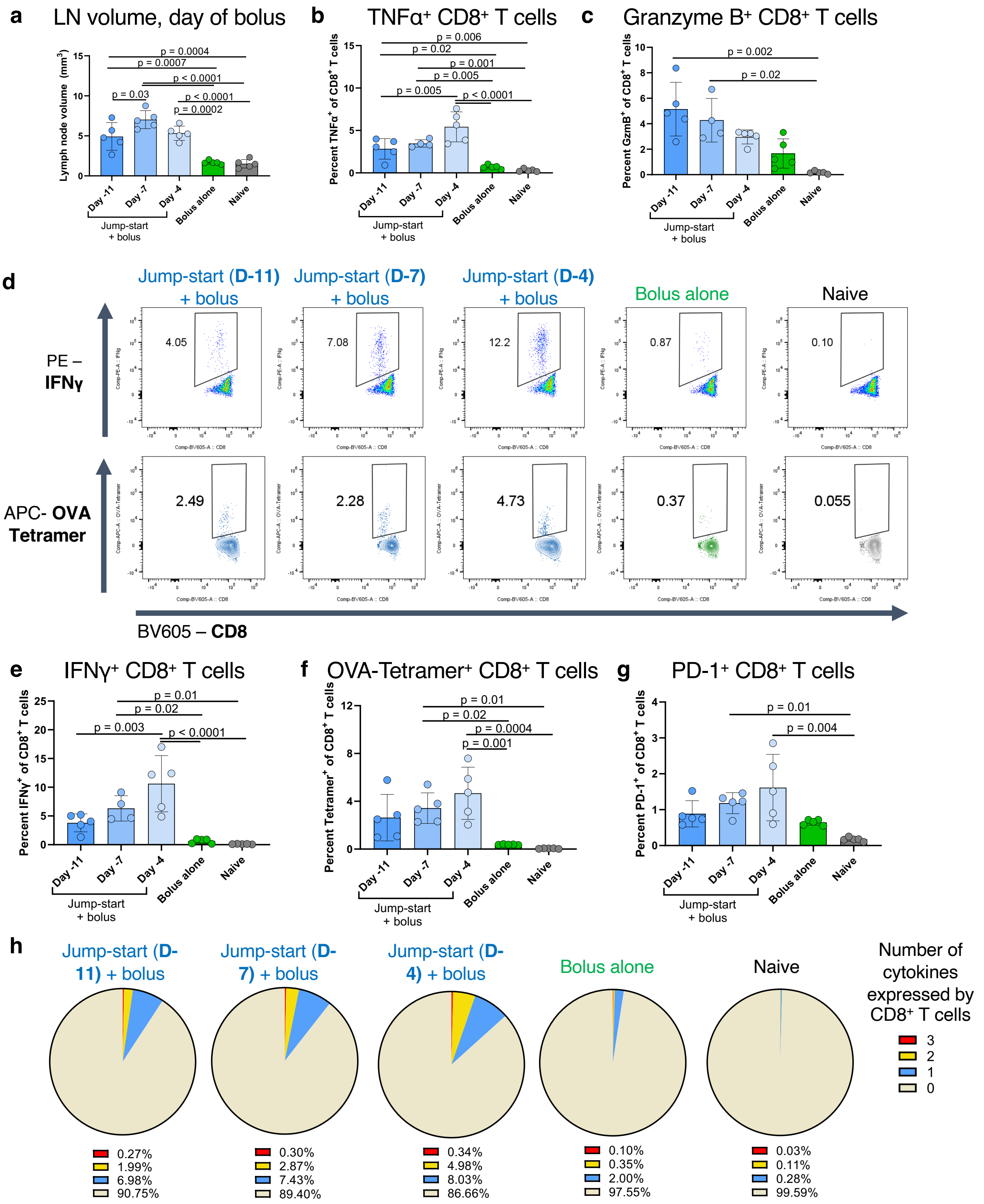

Supplementary Fig. 19 | Determining optimal timing of "jump-start" strategy to improve vaccine response. Mice were injected with an MPS no-antigen "jump-start" (GM-CSF and CpG) on days -11, -7, or -4, and then treated with a bolus vaccine (OVA protein, CpG, GM-CSF) on day 0. Control mice were treated with a bolus vaccine (no jump-start) on day 0 or left untreated (naïve). Draining inguinal lymph nodes were imaged on day 0, and mice were bled after 8 days for T cell analysis. (a) Day 0 lymph node volumes prior to bolus injection. Quantification of intracellular TNFα (b) and Granzyme B (c) in CD8+ T cells after ex vivo stimulation of PBMCs with OVA SIINFEKL peptide. (d) Representative flow cytometry plots depicting CD8+ T cell IFNγ production after restimulation with OVA SIINFEKL peptide (above) and OVA-tetramer binding (below). Quantification of (e) IFNγ production and (f) OVA-tetramer binding. (g) PD-1 expression on CD8+ T cells. (h) Pie charts depicting the percentage of CD8+ T cells expressing 0-3 of the cytokines IFNγ, TNFα, or Granzyme B in each group following OVA peptide restimulation. Tan represents T cells with no detectable cytokine expression, blue represents cells expressing a single cytokine, yellow represents two cytokines, and red represents cells expressing all three cytokines. For a, b, e, and f, statistical analysis was performed using

analysis of variance (ANOVA) with Tukey's post hoc test. For c and g, statistical analysis was performed using a Kruskal-Wallis test with Dunn's post hoc test. For a and f-g, n = 5 biologically independent animals per group; for b-e and h, n = 4 (Day -7 group) or 5 (all other groups) biologically independent animals per group. For a-c and e-g, means depicted; error bars, s.d.

a

Experimental groups

Group	Day -7	Day 0
Naïve	n/a	n/a
Bolus	n/a	Bolus vaccine
Jump-start/naïve	MPS, no ag	n/a
Jump-start/bolus	MPS, no ag	Bolus vaccine

b

Timeline

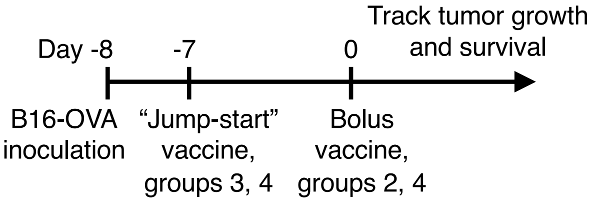

Supplementary Fig. 20. Therapeutic “jump-start” experiment layout. Mice bearing B16-OVA tumours (day -8) were treated at day -7 with an MPS “jump-start” (MPS material, GM-CSF, and CpG without antigen) or left untreated and were injected with a bolus vaccine (GM-CSF, CpG, and OVA) or left untreated. Tumour growth and survival were tracked. (a) Experimental groups. (b) Timeline.

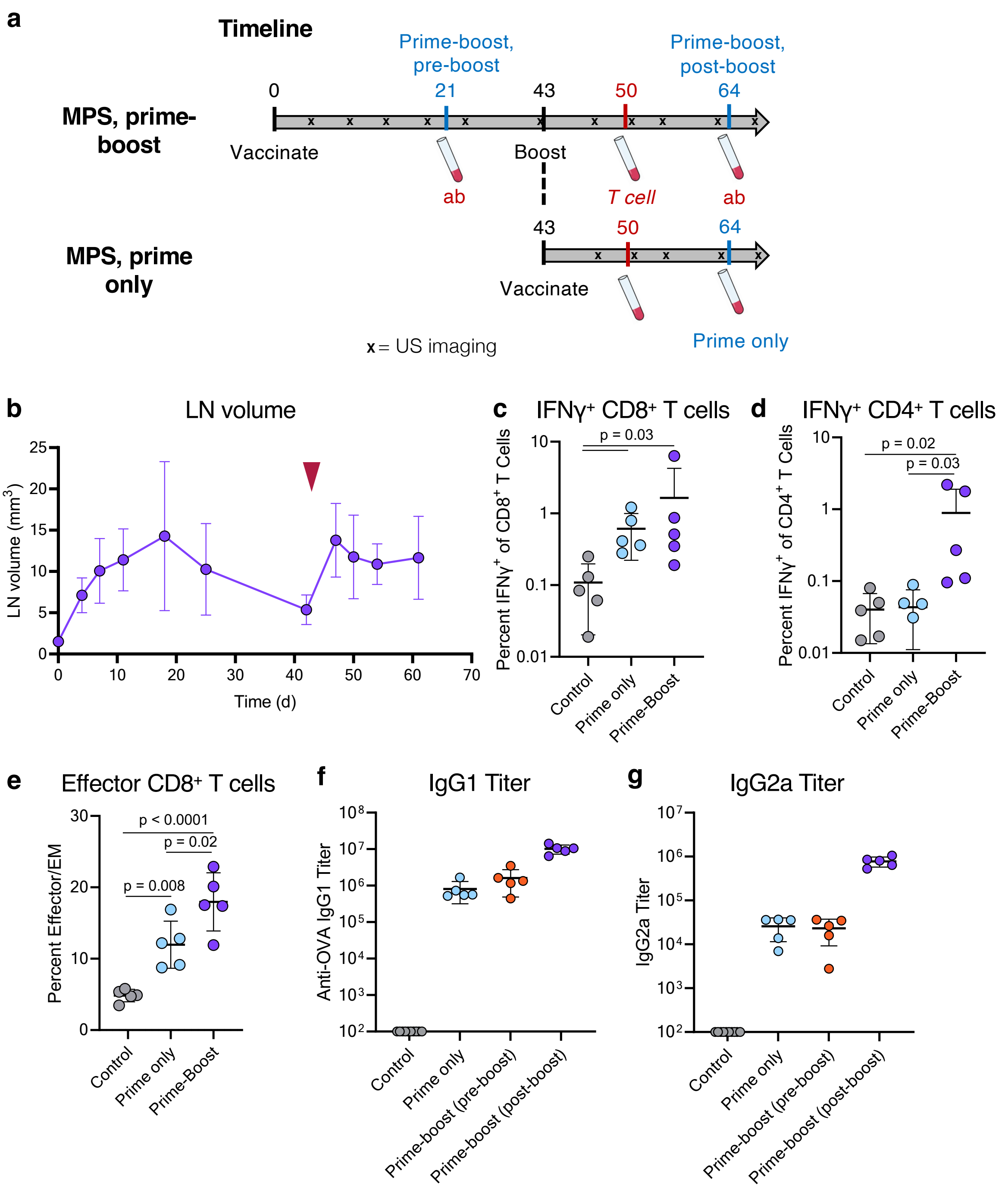

Supplementary Fig. 21 | Booster MPS vaccination expands lymph nodes and improves adaptive immune responses. (a) Timeline. Mice were immunized with MPS vaccines (GM-CSF, CpG, and OVA protein). Draining LNs were longitudinally imaged using HFUS. Once LNs had contracted in size, a booster MPS vaccine was injected adjacent to the same dLN on day 43. On the same day, a new group of mice were injected with an MPS vaccine ("prime only"). After 7 and 21 days, blood was collected for T cell and antibody analysis, respectively. (b) Lymph node volume over time. The red arrow indicates the timing of booster vaccination. n = 5 biologically independent animals. (c) Proportion of IFNγ-expressing $CD8^+$ T cells after stimulation with OVA peptides. (c) Proportion of IFNγ-expressing $CD4^+$ T cells after stimulation with OVA peptides. (e) Proportion of effector-type ($CD44^+CD62L^-$) $CD8^+$ T cells. IgG1 (f) and IgG2a (g) titers against OVA. For b-g, means depicted; error bars, s.d. For c-e, n = 5 biologically independent animals per group. For f-g, n = 10 (control) or 5 (all other groups) biologically independent animals per group.

Supplementary Table 1 | Antibodies utilized in flow cytometry.

Target	Fluorophore	Clone	Source	µL added per 100µL
B220	BV510	RA3-6B2	BioLegend	1.5
B220	BV570	RA3-6B2	BioLegend	2
CD3	APC/Fire 810	17A2	BioLegend	1.25
CD3	PerCP/Cy5.5	17A2	BioLegend	2
CD3	Pacific Blue	17A2	BioLegend	1.25
CD3	BV570	17A2	BioLegend	2
CD3	PE/Cy5	145-2C11	BioLegend	1.25
CD4	PerCP/Cy5.5	GK1.5	BioLegend	1
CD4	BV711	RM4-5	BioLegend	1.5
CD4	PE/Dazzle 594	RM4-5	BioLegend	1.25
CD8	BV 605	53-6.7	BioLegend	1.25
CD8	FITC	53-6.7	BioLegend	0.5
CD11b	PerCP/Cy5.5	M1/70	BioLegend	1
CD11b	BV421	M1/70	BioLegend	1
CD11c	PE/Cy5	N418	BioLegend	1.25
CD11c	PE/Cy7	N418	BioLegend	1
CD19	Spark NIR 685	6D5	BioLegend	1
CD24	BV421	M1/69	BioLegend	1
CD26	PE/Cy7	H194-112	BioLegend	1.8
CD31	FITC	MEC13.3	BioLegend	1.5
CD35/21 (CR1/2)	APC	7E9	BioLegend	1.5
CD44	BV 510	IM7	BioLegend	1.25
CD44	PE	IM7	BioLegend	1.25
CD44	APC/Fire 750	IM7	BioLegend	1.25
CD45	APC/Fire 750	30-F11	BioLegend	1
CD45	PerCP/Cy5.5	30-F11	BioLegend	1
CD45	PE/Cy7	30-F11	BioLegend	1
CD49b	PE/Dazzle 594	DX5	BioLegend	1
CD54 (ICAM-1)	Pacific Blue	YN1/1.7.4	BioLegend	1.5
CD62L	BV 785	MEL-14	BioLegend	1.25
CD62L	PE/Cy7	MEL-14	BioLegend	1.25
CD62L	FITC	MEL-14	BioLegend	1.25
CD64	BV 711	X54-5/7.1	BioLegend	1.8
CD68	PE	FA-11	BioLegend	1
CD103	BV785	2E7	BioLegend	2
CD301b	PE/Dazzle 594	URA-1	BioLegend	2
CLEC-2	PE	17D9	BioLegend	1.25
F4/80	APC/Fire 750	BM8	BioLegend	1
F4/80	APC	BM8	BioLegend	1
Gl7	Pacific Blue	GL7	BioLegend	0.5
Granzyme B	APC/Fire 750	QA16A02	BioLegend	5
IFNγ	APC	XMG1.2	BioLegend	1.5
IFNγ	PE	XMG1.2	BioLegend	1.5
IL-2	PE	JES6-5H4	BioLegend	3
Ly6C	AF 700	HK1.4	BioLegend	0.5
Ly6C	APC	HK1.4	BioLegend	1
Ly6G	BV785	1A8	BioLegend	1.5
Ly6G	BV570	1A8	BioLegend	2
Ly6G	PE/Cy7	1A8	BioLegend	1.5
MAdCAM1	PE	MECA-367	BioLegend	1.5
MHCII	FITC	M5/114.15.2	BioLegend	0.5
NK1.1	PE/Cy5	PK136	BioLegend	2
NK1.1	BV570	PK136	BioLegend	2
PD-1	PerCP/Cy5.5	RMP1-30	BioLegend	2

PD-1	BV510	29F.1A12	BioLegend	2
Podoplanin	PE/Cy7	8.1.1	BioLegend	2.5
Podoplanin	APC	8.1.1	BioLegend	1.25
Siglec H	PE	551	BioLegend	1
SIINFEKL-H-2Kb	PE/Dazzle 594	25-D1.16	BioLegend	2
TNFα	PE/Cy7	MP6-XT22	BioLegend	1.25

Supplementary Table 2 | Antibodies utilized in lymph node immunohistochemistry.

Target	Conjugate	Clone/Catalog #	Source	Dilution
B220	AF594	RA3-6B2	BioLegend	1:50
CCR2	n/a	EPR20844	Abcam	1:50
CD3	AF647	17A2	BioLegend	1:100
CD11b	AF488	M1/70	BioLegend	1:100
Collagen I	n/a	AB765P	Millipore Sigma	1:40
Collagen VI	AF488	ER-TR7	Santa Cruz Biotechnology	1:50
F-actin (phalloidin)	AF488	A12379	Thermo Fisher Scientific	1:100
Hyaluronic acid	Biotin	385911	Millipore Sigma	1:50
Ly6C	Biotin	ER-MP20	Abcam	1:200
