# Supplementary material for: Durable lymph-node expansion is associated with the efficacy of therapeutic vaccination
Source: Nat Biomed Eng. 2024 May 6;8(10):1226–42. doi: 10.1038/s41551-024-01209-3 (PMC11485260; doi:10.1038/s41551-024-01209-3)
Supplement: Supplementary file 2 — Reporting Summary [file 41551_2024_1209_MOESM2_ESM.pdf]

nature portfolio

Corresponding author(s): David J. Mooney

Last updated by author(s): Jan 6, 2024

Reporting Summary

Nature Portfolio wishes to improve the reproducibility of the work that we publish. This form provides structure for consistency and transparency in reporting. For further information on Nature Portfolio policies, see our Editorial Policies and the Editorial Policy Checklist.

Statistics

For all statistical analyses, confirm that the following items are present in the figure legend, table legend, main text, or Methods section.

n/a	Confirmed	
☐	☒	The exact sample size (*n*) for each experimental group/condition, given as a discrete number and unit of measurement
☐	☒	A statement on whether measurements were taken from distinct samples or whether the same sample was measured repeatedly
☐	☒	The statistical test(s) used AND whether they are one- or two-sided<br>*Only common tests should be described solely by name; describe more complex techniques in the Methods section.*
☒	☐	A description of all covariates tested
☐	☒	A description of any assumptions or corrections, such as tests of normality and adjustment for multiple comparisons
☐	☒	A full description of the statistical parameters including central tendency (e.g. means) or other basic estimates (e.g. regression coefficient) AND variation (e.g. standard deviation) or associated estimates of uncertainty (e.g. confidence intervals)
☐	☒	For null hypothesis testing, the test statistic (e.g. *F*, *t*, *r*) with confidence intervals, effect sizes, degrees of freedom and *P* value noted<br>*Give P values as exact values whenever suitable.*
☒	☐	For Bayesian analysis, information on the choice of priors and Markov chain Monte Carlo settings
☒	☐	For hierarchical and complex designs, identification of the appropriate level for tests and full reporting of outcomes
☒	☐	Estimates of effect sizes (e.g. Cohen's *d*, Pearson's *r*), indicating how they were calculated

Our web collection on statistics for biologists contains articles on many of the points above.

Software and code

Policy information about availability of computer code

Data collection	MATLAB (vR2020a, Mathworks) was used to quantify actin distribution in lymph nodes, SpectroFlo (v3.0, Cytek) was used for cytometry data acquisition, and Zen Black (v2.1, Zeiss) was used for imaging data collection. The custom code for quantifying lymph-node immunohistochemistry images is available at the Harvard Dataverse repository with the identifier https://doi.org/10.7910/DVN/BB8OSJ (ref. 64). For scRNA-seq, no new algorithms were developed for this project. Analysis code is available through the Open Science Framework (OSF) project with ID b5rcz (https://osf.io/b5rcz/?view_only=f15ba3fd86af40ddbb6ef6486f486a11).
Data analysis	FlowJo (v10.4) was used for flow-cytometry data analysis, VevoLab (v 5.6.1, VisualSonics) was used for volume quantification of lymph-node images, Prism (v9 and v10, Graphpad) were used for statistical analysis and plotting, ImageJ (v1.53m) was used for image analysis, and Adobe Illustrator (v22.1, Adobe) was used for generating maps of lymph-node mechanical properties.

For manuscripts utilizing custom algorithms or software that are central to the research but not yet described in published literature, software must be made available to editors and reviewers. We strongly encourage code deposition in a community repository (e.g. GitHub). See the Nature Portfolio guidelines for submitting code & software for further information.

Data

Policy information about availability of data

All manuscripts must include a data availability statement. This statement should provide the following information, where applicable:

- Accession codes, unique identifiers, or web links for publicly available datasets
- A description of any restrictions on data availability
- For clinical datasets or third party data, please ensure that the statement adheres to our policy

All datasets used in this study are included in the paper and its Supplementary information, and are available from the Harvard Dataverse repository with the identifier https://doi.org/10.7910/DVN/BB8OSJ (ref. 64). The scRNA-seq datasets generated during this study are available from the ArrayExpress database under accession code E-MTAB-13698 (https://www.ebi.ac.uk/biostudies/arrayexpress/studies/E-MTAB-13698) (ref. 65).

Research involving human participants, their data, or biological material

Policy information about studies with human participants or human data. See also policy information about sex, gender (identity/presentation), and sexual orientation and race, ethnicity and racism.

Reporting on sex and gender	The study did not involve human participants.
Reporting on race, ethnicity, or other socially relevant groupings	–
Population characteristics	–
Recruitment	–
Ethics oversight	–

Note that full information on the approval of the study protocol must also be provided in the manuscript.

Field-specific reporting

Please select the one below that is the best fit for your research. If you are not sure, read the appropriate sections before making your selection.

☒ Life sciences ☐ Behavioural & social sciences ☐ Ecological, evolutionary & environmental sciences

For a reference copy of the document with all sections, see nature.com/documents/nr-reporting-summary-flat.pdf

Life sciences study design

All studies must disclose on these points even when the disclosure is negative.

Sample size	Sample sizes for the in vivo experiments were determined empirically on the basis of results from prior publications, selecting appropriate numbers to achieve statistical significance while minimizing the number of mice used in accordance with the 3 Rs of animal use (Kim, J., et al. Nature Biotechnology 2015, doi: 10.1038/nbt.3071; Dellacherie, M., et al. Advanced Functional Materials, https://doi.org/10.1002/adfm.202002448) and with input and approval from Harvard University's Institutional Animal Care and Use Committee.
Data exclusions	In analysing nanoindentation data of lymph nodes, outlier data points (such as when the nanoindenter tip struck the sample mould rather than the sample itself) were excluded based on outlier analyses using GraphPad Prism v9.0. Mice with undetectable or inconsistent LN measurements (rare, 1–2 across hundreds in studies) were excluded from further analysis.
Replication	Replication information is included in the figure legends. Major experiments were conducted two to three times independently, to confirm results.
Randomization	The animals were randomized prior to initiation of the in vivo studies. In tumour studies, mouse tumours were measured prior to treatment and subsequently randomized into treatment groups.
Blinding	The majority of the data acquired were not blinded owing to quantifiable and unbiased outputs, feasibility and personnel limitations, or because the initial studies were exploratory. The repeat experiment confirming efficacy of the jump-start vaccine was performed in a blinded fashion.

Reporting for specific materials, systems and methods

We require information from authors about some types of materials, experimental systems and methods used in many studies. Here, indicate whether each material, system or method listed is relevant to your study. If you are not sure if a list item applies to your research, read the appropriate section before selecting a response.

Materials & experimental systems

n/a	Involved in the study	
☐	☒	Antibodies
☐	☒	Eukaryotic cell lines
☒	☐	Palaeontology and archaeology
☐	☒	Animals and other organisms
☒	☐	Clinical data
☒	☐	Dual use research of concern
☒	☐	Plants

Methods

n/a	Involved in the study	
☒	☐	ChIP-seq
☐	☒	Flow cytometry
☒	☐	MRI-based neuroimaging

Antibodies

Antibodies used

The following antibodies were used, and are listed in Supplementary Table 1 and Supplementary Table 2 of the manuscript:

B220 BV510 RA3-6B2 BioLegend 1.5
B220 BV570 RA3-6B2 BioLegend 2
CD3 APC/Fire 810 17A2 BioLegend 1.25
CD3 PerCP/Cy5.5 17A2 BioLegend 2
CD3 Pacific Blue 17A2 BioLegend 1.25
CD3 BV570 17A2 BioLegend 2
CD3 PE/Cy5 145-2C11 BioLegend 1.25
CD4 PerCP/Cy5.5 GK1.5 BioLegend 1
CD4 BV711 RM4-5 BioLegend 1.5
CD4 PE/Dazzle 594 RM4-5 BioLegend 1.25
CD8 BV 605 53-6.7 BioLegend 1.25
CD8 FITC 53-6.7 BioLegend 0.5
CD11b PerCP/Cy5.5 M1/70 BioLegend 1
CD11b BV421 M1/70 BioLegend 1
CD11c PE/Cy5 N418 BioLegend 1.25
CD11c PE/Cy7 N418 BioLegend 1
CD19 Spark NIR 685 6D5 BioLegend 1
CD24 BV421 M1/69 BioLegend 1
CD26 PE/Cy7 H194-112 BioLegend 1.8
CD31 FITC MEC13.3 BioLegend 1.5
CD35/21 (CR1/2) APC 7E9 BioLegend 1.5
CD44 BV 510 IM7 BioLegend 1.25
CD44 PE IM7 BioLegend 1.25
CD44 APC/Fire 750 IM7 BioLegend 1.25
CD45 APC/Fire 750 30-F11 BioLegend 1
CD45 PerCP/Cy5.5 30-F11 BioLegend 1
CD45 PE/Cy7 30-F11 BioLegend 1
CD49b PE/Dazzle 594 DX5 BioLegend 1
CD54 (ICAM-1) Pacific Blue YN1/1.7.4 BioLegend 1.5
CD62L BV 785 MEL-14 BioLegend 1.25
CD62L PE/Cy7 MEL-14 BioLegend 1.25
CD62L FITC MEL-14 BioLegend 1.25
CD64 BV 711 X54-5/7.1 BioLegend 1.8
CD68 PE FA-11 BioLegend 1
CD103 BV785 2E7 BioLegend 2
CD301b PE/Dazzle 594 URA-1 BioLegend 2
CLEC-2 PE 17D9 BioLegend 1.25
F4/80 APC/Fire 750 BM8 BioLegend 1
F4/80 APC BM8 BioLegend 1
Gl7 Pacific Blue GL7 BioLegend 0.5
Granzyme B APC/Fire 750 QA16A02 BioLegend 5
IFNγ APC XMG1.2 BioLegend 1.5
IFNγ PE XMG1.2 BioLegend 1.5
IL-2 PE JES6-5H4 BioLegend 3
Ly6C AF 700 HK1.4 BioLegend 0.5
Ly6C APC HK1.4 BioLegend 1
Ly6G BV785 1A8 BioLegend 1.5
Ly6G BV570 1A8 BioLegend 2
Ly6G PE/Cy7 1A8 BioLegend 1.5
MAdCAM1 PE MECA-367 BioLegend 1.5
MHCII FITC M5/114.15.2 BioLegend 0.5
NK1.1 PE/Cy5 PK136 BioLegend 2
NK1.1 BV570 PK136 BioLegend 2

PD-1 PerCP/Cy5.5 RMP1-30 BioLegend 2
PD-1 BV510 29F.1A12 BioLegend 2
Podoplanin PE/Cy7 8.1.1 BioLegend 2.5
Podoplanin APC 8.1.1 BioLegend 1.25
Siglec H PE 551 BioLegend 1
SIINFEKL-H-2Kb PE/Dazzle 594 25-D1.16 BioLegend 2
TNFα PE/Cy7 MP6-XT22 BioLegend 1.25

B220 AF594 RA3-6B2 BioLegend 1:50
CCR2 n/a EPR20844 Abcam 1:50
CD3 AF647 17A2 BioLegend 1:100
CD11b AF488 M1/70 BioLegend 1:100
Collagen I n/a AB765P Millipore Sigma 1:40
Collagen VI AF488 ER-TR7 Santa Cruz Biotechnology 1:50
F-actin (phalloidin) AF488 A12379 Thermo Fisher Scientific 1:100
Hyaluronic acid Biotin 385911 Millipore Sigma 1:50
Ly6C Biotin ER-MP20 Abcam 1:200

Validation

Antibodies were used according to manufacturer recommendations and gated based on known negative (such as FMO or secondary only) controls. At first use, the antibody volumes used in experiments were tested on control mouse samples to verify reactivity. The validation statement included on the manufacturer's (BioLegend's) website, states "Each lot of this antibody is quality control tested by intracellular immunofluorescent staining with flow cytometric analysis. For flow cytometric staining, the suggested use of this reagent is ≤0.25 µg per million cells in 100 µl volume. It is recommended that the reagent be titrated for optimal performance for each application." Initial antibody volume choices were based on this recommendation.

Eukaryotic cell lines

Policy information about cell lines and Sex and Gender in Research

Cell line source(s)

The B16-OVA cell line was obtained from Kai Wucherpfennig's laboratory (Dana Farber Cancer Institute, Boston, MA). This cell line is derived from the commercially available B16-F10 line (ATCC) and can be purchased commercially from Biocytogen.

Authentication

The cell line was not authenticated.

Mycoplasma contamination

The cell line was not tested for mycoplasma contamination.

Commonly misidentified lines
(See ICLAC register)

No commonly misidentified cell lines were used.

Animals and other research organisms

Policy information about studies involving animals; ARRIVE guidelines recommended for reporting animal research, and Sex and Gender in Research

Laboratory animals

Female C57BL/6J mice, aged 6–8 weeks on initiation of each study, were purchased from Jackson Laboratory (Bar Harbor, ME). Mice were housed with food and water ad libitum, and light was provided in 14-h-light–10-h-dark cycles. Mice were housed at ambient temperature 22 deg (+/– 1 deg) at 30–70% humidity.

Wild animals

The study did not involve wild animals.

Reporting on sex

Female mice were used in all experiments.

Field-collected samples

The study did not involve samples collected from the field.

Ethics oversight

All animal procedures were compliant with relevant ethical regulations established by the National Institutes of Health and institutional guidelines with the approval of Harvard University's Institutional Animal Care and Use Committee.

Note that full information on the approval of the study protocol must also be provided in the manuscript.

Flow Cytometry

Plots

Confirm that:

☒ The axis labels state the marker and fluorochrome used (e.g. CD4-FITC).

☒ The axis scales are clearly visible. Include numbers along axes only for bottom left plot of group (a 'group' is an analysis of identical markers).

☒ All plots are contour plots with outliers or pseudocolor plots.

☒ A numerical value for number of cells or percentage (with statistics) is provided.

Methodology

Sample preparation	Blood was collected from mice retro-orbitally using heparinized capillary tubes (Fisherbrand) and stored in heparinized collection tubes (BD Biosciences) on ice. Red blood cells were lysed using ACK Lysing Buffer (Quality Biological). LNs were explanted and digested in RPMI-1640 (Corning) containing 0.8mg/mL Dispase II, 0.2mg/mL Collagenase P, and 0.1mg/mL DNase I (all Roche, procured from Sigma) until no visible LN pieces remained, following established protocols.
Instrument	Samples were run on an Aurora Spectral Analyzer (Cytek) or LSRII flow cytometer (BD Biosciences).
Software	Data were acquired using SpectroFlo software (Cytek) and analysed using Flowjo v10 software.
Cell population abundance	Abundance of cell populations within flow-cytometry results are specified throughout the manuscript in reference to specific experiments.
Gating strategy	Cells were gated on (1) SSC-A vs. FSC-A, selecting the major cell population in accordance with published datasets, (2) fSC-H vs. FSC-A, selecting single cells based on the visible population distribution, (3) live cells based on negative staining for a viability dye, and then (4) subsequent stains dependent on the panel of interest. Sample gating strategies are included throughout the manuscript and particularly in Supplementary Fig. 7.

☒ Tick this box to confirm that a figure exemplifying the gating strategy is provided in the Supplementary Information.
